# Comparative efficacy of postoperative adjuvant transcatheter arterial chemoembolization versus lenvatinib plus tislelizumab in patients with BCLC stage 0 -B hepatocellular carcinoma after radical resection

**DOI:** 10.3389/fimmu.2026.1778330

**Published:** 2026-06-29

**Authors:** Xu Feng, Yupei Ao, Lili Wang, Kai Chen, Chengjia Tang, Chengcheng Huang

**Affiliations:** 1 Department of Hepatobiliary Surgery, The Affiliated Yongchuan Hospital of Chongqing Medical University, Chongqing, China; 2 Department of Hepatobiliary Surgery, The First Affiliated Hospital of Chongqing Medical University, Chongqing, China; 3 Health Screening Centre, Chongqing Western Hospital, Chongqing, China; 4 Department of Geriatrics, The Affiliated Yongchuan Hospital of Chongqing Medical University, Chongqing, China

**Keywords:** hepatocellular carcinoma, postoperative adjuvant, radical liver resection, targeted therapy and immunotherapy, transcatheter arterial chemoembolization

## Abstract

**Aim:**

This study aimed to compare the efficacy of postoperative adjuvant transcatheter arterial chemoembolization (PA-TACE) and adjuvant lenvatinib plus tislelizumab (PA-LT) in patients with Barcelona Clinic Liver Cancer (BCLC) stage 0–B hepatocellular carcinoma (HCC) who were at high risk of recurrence following radical liver resection (LR).

**Methods:**

This study retrospectively evaluated HCC patients who underwent LR at two clinical centers between January 1, 2019 and January 31, 2025. Recurrence-free survival (RFS) and overall survival (OS) were compared across three groups: LR alone, PA-TACE, and PA-LT. Propensity score matching (PSM) was conducted to reduce intergroup heterogeneity and to enhance the robustness of the results.

**Results:**

A total of 614 patients with HCC at high risk of recurrence after radical resection were included in this study. After PSM, 148, 223, and 75 patients were retained in the LR, PA-TACE, and PA-LT groups, respectively. Compared with the LR group, both the PA-TACE and PA-LT groups were associated with significant improvements in RFS and OS (all p < 0.001), and no severe adverse events were observed in either group. However, no significant differences in RFS or OS were observed between the PA-TACE and PA-LT groups (median RFS: 44.00 months (95% CI, 40.91–47.09) vs. 40.50 months (95% CI, 34.90–46.10), p = 0.086; median OS: 69.00 months (95% CI, 66.44–71.56) vs. 65.00 months (95% CI, 60.93–69.07), p = 0.572). Multivariable Cox regression analysis demonstrated that both PA-TACE and PA-LT were independently associated with improved RFS and OS.

**Conclusion:**

Both PA-TACE and PA-LT were associated with improved RFS and OS in hepatocellular carcinoma patients at high risk of recurrence after radical resection, with acceptable safety profiles. However, no significant differences were observed between the two adjuvant treatment strategies.

## 1 Introduction

Primary liver cancer is the sixth most common cancer globally and the second leading cause of cancer-related deaths, with a particularly high prevalence in resource-limited developing countries. Hepatocellular carcinoma (HCC) accounts for approximately 75%–85% of all primary liver cancer cases (1). In China, HCC is the fifth most common cancer and the second leading cause of cancer-related deaths (2). For HCC treatment, radical methods such as ablation, radical liver resection (LR), and liver transplantation are the primary choices. However, due to the inherent heterogeneity of HCC, there still remains a high recurrence rate even after radical resection. According to statistics, the recurrence rate within 5 years after radical resection remains as high as 50%–70%. Compared to patients without recurrence, patients with HCC recurrence had an approximately 24% lower 5-year survival rate (3–5).

It is widely recognized that multiple tumors, tumor diameter ≥ 5 cm, vascular invasion, and poor differentiation are high-risk recurrence factors (HRRFs) following radical resection of HCC (6–8). Previous studies have shown that the 1-year recurrence rates in patients with microvascular invasion (MVI), tumors measuring 5–10 cm, and tumors measuring ≥ 10 cm approach 50%, 37%, and 57%, respectively, whereas the 6-month recurrence rate in patients with multiple tumors is as high as 60%. In addition, the 5-year survival rates reported for patients with MVI, multiple tumors, tumors measuring 5–10 cm, and tumors measuring ≥ 10 cm are 33.3%, 31.9%, 62.3%, and 53.2%, respectively (9–11).

Postoperative adjuvant transcatheter arterial chemoembolization (PA-TACE) has become a widely adopted adjuvant treatment strategy for HCC patients with HRRFs, and accumulating evidence suggests that PA-TACE is associated with significant improvements in survival outcomes (12–14). Similarly, consistent findings have shown that targeted therapy combined with immunotherapy improves outcomes in HCC patients with HRRFs (15–17). However, these studies involved various combinations of targeted therapy and immunotherapy regimens, which may introduce inter-study heterogeneity and potentially affect the overall findings. Lenvatinib is a multi-target receptor tyrosine kinase inhibitor (TKI) that exerts antitumor effects by inhibiting key signaling pathways involved in tumor angiogenesis and cell proliferation, including vascular endothelial growth factor receptors and fibroblast growth factor receptors (18). Tislelizumab, an immune checkpoint inhibitor targeting PD-1, has shown promise in enhancing the immune response against tumor cells (19). In unresectable HCC, the combination of lenvatinib and tislelizumab has demonstrated promising antitumor activity along with a favorable safety profile (20, 21). In our retrospective study, we also demonstrated that postoperative adjuvant lenvatinib plus tislelizumab (PA-LT) conferred significant benefits in patients with HCC after radical resection, with a relatively low incidence of adverse events (22).

To date, there are limited studies specifically comparing PA-TACE with PA-LT. Therefore, in the present study, we aimed to compare the efficacy of PA-TACE versus PA-LT in patients with HCC.

## 2 Materials and methods

### 2.1 Patients

This retrospective study systematically evaluated patients with HCC who underwent LR between January 1, 2019, and January 31, 2025 in the Department of Hepatobiliary Surgery of the First Affiliated Hospital of Chongqing Medical University and the Department of Hepatobiliary Surgery of the Affiliated Yongchuan Hospital of Chongqing Medical University. All consecutive eligible cases were included. The study was conducted in accordance with the Declaration of Helsinki and was approved by the ethics committees of the two participating centers. As this was a retrospective study, the requirement for informed consent was waived.

Eligible patients were screened according to the following inclusion criteria: 1) histologically confirmed HCC with negative margins (R0 resection); 2) at least one recurrence risk factor, including multiple tumors, tumor diameter ≥ 5 cm, poor histological differentiation, or MVI; 3) Barcelona Clinic Liver Cancer (BCLC) stage 0–B; 4) preserved liver function classified as Child–Pugh class A (score 5–6); 5) received either no postoperative adjuvant therapy, PA-TACE, or PA-LT; 6) no antitumor therapy before resection; and 7) no history of other malignancies. Exclusion criteria: 1) incomplete clinical data or follow-up information; 2) non-radical resection (R1 resection) or preoperative imaging indicating portal vein invasion or extrahepatic metastasis; 3) postoperative pathological confirmation of non-HCC; 4) with other malignancies (including those in sustained remission) or severe comorbid conditions involving major organs such as the brain, heart, or lungs; 5) received postoperative adjuvant therapies other than TACE or LT; 6) treatment crossover between PA-TACE and PA-LT; 7) history of HCC rupture with associated hemorrhage; 8) the interval from LR to initiation of PA-TACE or PA-LT exceeded 6 weeks; 9) recurrence or death within 60 days postoperatively.

### 2.2 Postoperative adjuvant treatment strategies

Postoperative adjuvant therapy was initiated 4–6 weeks after LR. Prior to treatment initiation, patients underwent reassessment including serum alpha-fetoprotein (AFP) measurement and contrast-enhanced upper abdominal computed tomography (CT) or magnetic resonance imaging (MRI). In the absence of radiological evidence of tumor recurrence, adjuvant treatment recommendations were made by attending physicians with the rank of associate chief physician or higher, based on the patient’s overall clinical condition; however, the final decision was made in accordance with the patient’s preference. Meanwhile, all patients with hepatitis B virus (HBV) infection require lifelong antiviral therapy.

PA-TACE: A hepatic artery catheter was inserted via the femoral artery using the Seldinger technique, and the presence of intrahepatic tumor staining was evaluated by digital subtraction angiography. In the absence of tumor staining, chemotherapy drugs (one or more of the following: fluorouracil, anthracyclines, or platinum-based agents) and embolic agents (like lipiodol and/or gelatin sponge) are administered via the catheter to the liver segment containing the tumor, based on comprehensive evaluation of the patient’s body surface area, physical condition, and residual liver volume. The interval between treatments was typically 4–5 weeks (23). All PA-TACE sessions included in this study were performed as adjuvant therapy before radiological recurrence and in the absence of residual disease on postoperative imaging examinations. Repeated PA-TACE was selectively administered in patients considered at high risk of recurrence and with adequate liver function reserve, based on multidisciplinary evaluation. TACE performed for suspected or confirmed tumor recurrence during follow-up was not classified as PA-TACE.

PA-LT: Patients received intravenous tislelizumab (200 mg, day 1 of a 21-day cycle) combined with oral lenvatinib (12 mg daily for body weight ≥ 60 kg or 8 mg daily for < 60 kg). The dosing regimen was based on a phase II study demonstrating a manageable safety profile and was consistent with the prescribing information for both agents (20). Detailed information regarding all drugs used in this study is provided in the **Supplementary Table 1**.

During treatment, adverse events were recorded in detail according to the Common Terminology Criteria for Adverse Events (CTCAE), version 5.0 (24). If patients developed intolerable adverse events or grade 3/4 treatment-related adverse events (AEs) as defined by CTCAE, symptomatic management was provided, and the doses of chemotherapeutic and targeted/immunotherapeutic agents were reduced to 50% of the original dose when considered necessary, with temporary interruption or permanent discontinuation if required. After symptom resolution, patients were reassessed for eligibility to continue treatment.

### 2.3 Follow up and outcomes

All patients were followed up every 1–2 months for 6 months after discharge from the hospital and every 3–6 months thereafter. During the follow-up period, each patient received routine blood tests, liver function tests, AFP, and abdominal ultrasound. If recurrence was suspected, enhanced CT or enhanced MRI was used to confirm the diagnosis. Recurrence was defined as any tumor nodule confirmed by two imaging modalities or by percutaneous biopsy. The primary endpoint of the study was recurrence-free survival (RFS), defined as the time from LR to tumor recurrence or the end of follow-up, whichever occurred first. The secondary endpoint was overall survival (OS), defined as the time from LR to death from any cause or the end of follow-up, whichever occurred first. All patients were followed prospectively until December 1, 2025, or death, whichever occurred first.

### 2.4 Propensity score matching

Propensity score matching (PSM) analysis was conducted to minimize covariate imbalance among the three groups. A nearest-neighbor greedy matching algorithm without replacement was applied at a matching ratio of LR: PA-TACE: PA-LT = 2: 3: 1, using a caliper width of 0.10. Propensity scores were estimated via a multinomial logistic regression model incorporating the following covariates: age, gender, hepatitis status, presence of cirrhosis, AFP level, tumor number, tumor size, MVI, Edmondson–Steiner (ES) grade, resection pattern, intraoperative blood transfusion, and ALBI grade. Covariate balance was assessed using standardized mean differences (SMDs), with an SMD < 0.10 considered indicative of adequate balance, which is a commonly accepted threshold for good covariate balance in PSM analyses. Following matching, inverse probability of treatment weighting (IPTW) was additionally performed as a sensitivity analysis to evaluate the robustness of the results.

### 2.5 Statistical analysis

The Shapiro–Wilk test was used to assess the normality of continuous variables. For comparisons among three independent groups, variables with a normal distribution and homogeneity of variance were analyzed using one-way analysis of variance and presented as mean ± standard deviation. Variables that did not follow a normal distribution or showed heterogeneity of variance were analyzed using the Kruskal–Wallis test and expressed as median (interquartile range, IQR). Homogeneity of variance was assessed using Levene’s test. Categorical variables were presented as frequencies (n) and percentages (%), and comparisons between groups were performed using the chi-square test. Continuous variables were further dichotomized according to their median values or commonly used clinical cut-off thresholds and subsequently included in regression analyses. Fisher’s exact test was used when the expected cell count was less than 5. Survival analysis was performed using the Kaplan–Meier method, and differences between survival curves were compared using the log-rank test. Cox proportional hazards regression models were used to identify factors associated with survival outcomes. Univariate Cox regression analysis was first conducted, and variables with P < 0.05 were entered into the multivariable Cox regression model. Results were reported as hazard ratios (HRs) with 95% confidence intervals (CIs). The proportional hazards assumption of the Cox model was assessed using Schoenfeld residuals. All statistical analyses were two-sided, with a significance level of α = 0.05. Statistical analyses were performed using SPSS software (version 27.0) and R software (version 4.5.0).

## 3 Results

### 3.1 Baseline patient characteristics

**Figure 1:**
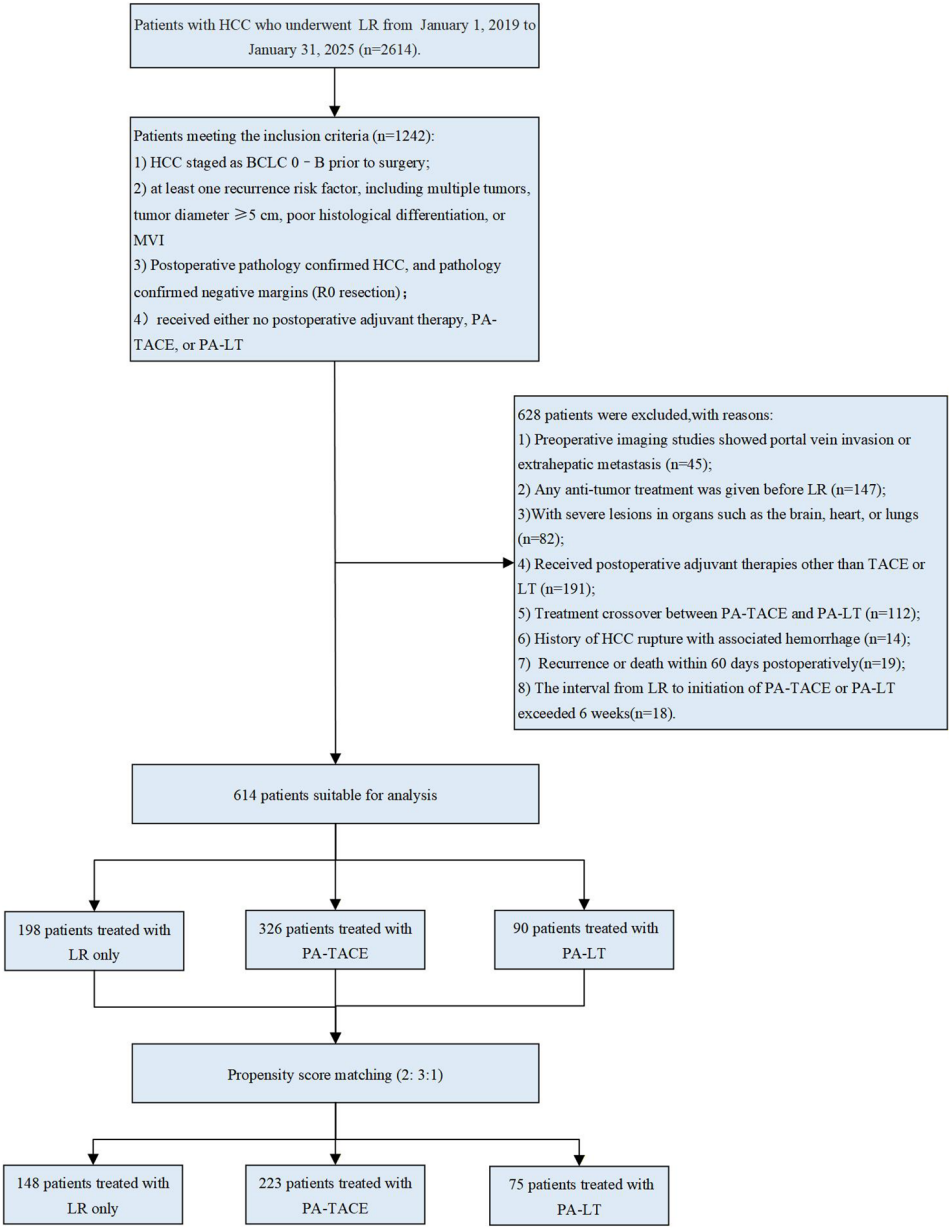
Flowchart of patient selection. HCC, hepatocellular carcinoma; LR, liver resection; BCLC, Barcelona Clinic Liver Cancer; MVI, microvascular invasion; PA, postoperative adjuvant; TACE, transcatheter arterial chemoembolization; LT, lenvatinib plus tislelizumab.

After screening, 614 eligible patients were included, comprising 198 in the LR group, 326 in the PA-TACE group, and 90 in the PA-LT group. After PSM, 148, 223, and 75 patients remained in the LR, PA-TACE, and PA-LT groups, respectively. The study flowchart is shown in **Figure 1**.

In the entire cohort, the mean age was 56.28 ± 9.96 years, and 532 patients (86.64%) were male. A total of 562 patients (91.53%) had BCLC stage 0/A, while 52 patients (8.47%) had BCLC stage B. Significant intergroup differences were observed only in intraoperative blood transfusion (p = 0.007), with no other baseline characteristics showing statistically significant differences. In the PSM cohort, the mean age was 56.21 ± 9.78 years, and 395 patients (88.57%) were male. A total of 412 patients (92.38%) had BCLC stage 0/A, while 34 patients (7.62%) had BCLC stage B. Baseline characteristics of both cohorts are summarized in **Table 1**.

**Table 1:** Baseline characteristics of HCC patients with HRRFs after LR.

Characteristics		The entire cohort					The PSM cohort				
		LR (n=198)	PA-TACE (n=326)	PA-LT (n=90)	P	SMD	LR (n=148)	PA-TACE (n=223)	PA-LT (n=75)	P	SMD
Age, years		56.79 ± 10.67	55.63 ± 9.50	57.52 ± 10.04	0.190	0.127	55.92 ± 10.52	56.30 ± 9.28	56.55 ± 9.84	0.889	0.042
Age, n (%)	≤ 56 yrs	100 (50.51)	183 (56.13)	42 (46.67)	0.199	0.127	77 (52.03)	120 (53.81)	41 (54.67)	0.916	0.035
	> 56 yrs	98 (49.49)	143 (43.87)	48 (53.33)			71 (47.97)	103 (46.19)	34 (45.33)		
Gender, n (%)	Male	174 (87.88)	278 (85.28)	80 (88.89)	0.554	0.072	129 (87.16)	200 (89.69)	66 (88.00)	0.745	0.053
	Female	24 (12.12)	48 (14.72)	10 (11.11)			9 (12.84)	23 (10.31)	9 (12.00)		
Hepatitis, n (%)	HBV	149 (75.25)	254 (77.91)	78 (86.67)	0.381	0.270	128 (86.48)	188 (84.31)	65 (86.66)	0.887	0.079
	HCV	17 (8.59)	27 (8.28)	7 (7.78)			12 (8.11)	19 (8.52)	6 (8.00)		
	AH	8 (4.04)	13 (3.99)	3 (3.33)			3 (2.03)	8 (3.59)	2 (2.67)		
	No	24 (12.12)	32 (9.82)	2 (2.22)			5 (3.38)	8 (3.59)	2 (2.67)		
Liver Cirrhosis yes, n (%)		146 (73.73)	216 (66.26)	63 (70.00)	0.195	0.109	112 (75.68)	163 (73.091)	59 (78.67)	0.607	0.087
AFP, n (%)	< 200 ng/ml	131 (66.17)	202 (61.96)	56 (62.22)	0.608	0.058	99 (66.89)	143 (64.13)	47 (62.67)	0.787	0.059
	≥ 200 ng/ml	67 (33.83)	124 (38.04)	34 (37.78)			49 (33.11)	80 (35.87)	28 (37.33)		
Tumor diameter, cm		4.30 (2.62, 6.00)	4.20 (2.80, 6.50)	3.65 (2.50, 5.60)	0.143	0.196	3.95 (2.50, 5.73)	4.00 (2.50, 5.80)	3.90 (2.50, 5.70)	0.782	0.094
Tumor diameter, n (%)	< 5cm	108 (54.55)	175 (53.68)	55 (61.11)	0.449	0.100	84 (56.76)	136 (60.99)	46 (61.33)	0.681	0.062
	≥ 5cm	90 (45.45)	151 (46.32)	35 (38.89)			64 (43.24)	87 (39.01)	29 (38.67)		
Tumor number, n (%)	Solitary	161 (81.31)	256 (78.53)	71 (78.89)	0.738	0.046	119 (80.41)	179 (80.27)	58 (77.33)	0.841	0.050
	Multiple	37 (18.69)	70 (21.47)	19 (21.11)			29 (19.59)	44 (19.73)	17 (22.67)		
BCLC stage, n (%)	0+A	188 (94.95)	292 (89.57)	82 (91.11)	0.099	0.135	139 (93.92)	205 (91.93)	68 (90.67)	0.646	0.082
	B	10 (5.05)	34 (10.43)	8 (8.89)			9 (6.08)	18 (8.07)	7 (9.33)		
ES grade, *n* (%)	3/4	47 (23.74)	86 (26.38)	21 (23.33)	0.730	0.047	33 (22.30)	51 (22.87)	17 (22.67)	0.992	0.009
	1/2	151 (76.26)	240 (73.62)	69 (76.67)			115 (77.70)	172 (77.13)	58 (77.33)		
MVI, *n* (%)	Positive	113 (57.07)	193 (59.20)	46 (51.11)	0.338	0.109	82 (55.40)	115 (51.57)	39 (52.00)	0.757	0.051
	Negative	85 (42.93)	133 (40.80)	44 (48.89)			66 (44.60)	108 (48.43)	36 (48.00)		
ALBI grade, n (%)	1	129 (65.15)	225 (69.02)	69 (76.67)	0.147	0.170	107 (72.30)	164 (73.54)	58 (77.33)	0.718	0.077
	2	69 (34.85)	101 (30.98)	21 (23.33)			41 (27.70)	59 (26.46)	17 (22.67)		
Child-Pugh score, n (%)	5	166 (83.84)	290 (88.96)	79 (87.78)	0.232	0.100	129 (87.16)	204 (91.48)	67 (89.33)	0.406	0.094
	6	32 (16.16)	36 (11.04)	11 (12.22)			19 (12.84)	19 (8.52)	8 (10.67)		
Resection pattern, n (%)	Anatomic	148 (74.75)	230 (70.55)	60 (66.77)	0.335	0.119	106 (71.62)	155 (69.51)	53 (70.67)	0.908	0.031
	Nonanatomic	50 (25.25)	96 (29.45)	30 (33.33)			42 (28.38)	68 (30.49)	22 (29.33)		
Blood transfusion yes, n (%)		35 (17.68)	43 (13.19)	7 (7.78)	0.007	0.201	14 (9.46)	24 (10.76)	7 (9.33)	0.894	0.032
Blood loss, ml		200.00 (100.00, 400.00)	230.00 (150.00, 400.00)	200.00 (100.00, 390.00)	0.568	0.118	200.00 (100.00, 400.00)	200.00 (150.00, 400.00)	200.00 (100.00, 372.50)	0.589	0.037
Operating time, minutes		240.00 (186.25, 310.00)	260.00 (195.00, 320.00)	250.00 (201.25, 300.00)	0.211	0.140	257.50 (195.00, 316.25)	260.00 (195.00, 317.00)	254.00 (202.50, 304.50)	0.979	0.094
*Hemoglobin, g/L		137.09 ± 21.32	140.72 ± 17.80	139.49 ± 21.71	0.121	0.119	140.12 ± 19.98	142.83 ± 15.63	140.77 ± 20.95	0.340	0.098
*Platelet, 10^9^/L		146.00 (108.00, 185.00)	151.00 (114.25, 202.00)	141.50 (102.25, 196.75)	0.141	0.135	143.50 (102.00, 185.00)	148.00 (111.00, 193.00)	138.00 (99.50, 195.00)	0.638	0.067
*Total protein, g/L		70.02 ± 6.75	70.75 ± 6.70	70.17 ± 7.78	0.460	0.070	70.45 ± 6.13	70.73 ± 6.23	70.15 ± 6.08	0.763	0.063
*ALT, U/L		32.50 (23.00, 47.00)	34.00 (24.00, 46.00)	31.75 (21.25, 45.75)	0.576	0.063	33.00 (23.00, 47.00)	35.00 (25.00, 47.50)	32.00 (21.00, 46.500)	0.444	0.086
*ALP, U/L		88.00 (69.25, 110.50)	88.00 (70.25, 110.00)	82.00 (67.00, 109.00)	0.671	0.066	88.00 (68.75, 108.00)	86.00 (70.00, 109.50)	86.00 (67.00, 108.00)	0.887	0.013
*γ-GGT, U/L		54.00 (30.00, 111.75)	56.00 (34.00, 104.25)	52.00 (30.00, 83.50)	0.581	0.118	51.50 (30.75, 100.00)	51.00 (34.00, 97.00)	52.00 (34.00, 94.00)	0.850	0.099
Resection margin ≥ 1cm, n (%)		198 (100.00)	326 (100.00)	90 (100.00)	1.000	–	148 (100.00)	223 (100.00)	75 (100.00)	1.000	–

PSM, propensity score matching; LR, liver resection; PA, postoperative adjuvant; TACE, transcatheter arterial chemoembolization; LT, lenvatinib plus tislelizumab; SMD, standardized mean differences; HBV, hepatitis B virus; HCV, hepatitis C virus; AH, alcoholic hepatitis; AFP, alpha-fetoprotein; BCLC, Barcelona Clinic Liver Cancer. ES, Edmondson-Steiner; MVI, microvascular invasion; ALBI, albumin-bilirubin; ALBl = (log10 total bilirubin*0.66) + (albumin * -0.085); ALBI grade 1, ≤ -2.60; ALBI grade 2, -2.60 ~ -1.39; ALT, aspartate aminotransferase; ALP, alkaline phosphatase; γ-GGT, Gamma-glutamyl transferase.

ES, grade 1, well-differentiated tumors, grade 2, moderately differentiated tumors; grade 3, poorly differentiated tumors, and grade 4, undifferentiated or highly poorly differentiated tumors; MVI, microvascular invasion, was diagnosed when cancer cell clusters were observed within vascular lumina lined by endothelial cells, typically in small branches of the portal vein in the peritumoral liver tissue or in blood vessels within the tumor capsule. In this study, all pathological assessments were performed independently by two experienced pathologists based on postoperative specimens. In cases of disagreement, a consensus was reached through joint review and discussion.

Anatomical hepatectomy is the complete resection of the segment of liver with the tumor or the segment of liver limited by the branches of the portal vein of the tumor; Non-anatomical hepatectomy is the resection of the tumor and part of the non-tumor liver parenchyma. All surgical procedures were performed by experienced hepatobiliary surgeons with senior professional titles (associate chief physician or higher), each with extensive experience in hepatic resection.

*All laboratory parameters were measured preoperatively.

### 3.2 Recurrence-free survival time

In the entire cohort, the median follow-up time was 46.00 months (95% CI, 44.55–47.95). Recurrence occurred in 164 patients (82.83%) in the LR group, 205 (62.88%) in the PA-TACE group, and 43 (47.78%) in the PA-LT group. The median RFS (mRFS) was 26.00 months (95% CI, 25.03–26.97) in the LR group, 42.00 months (95% CI, 39.76–44.24) in the PA-TACE group, and 39.50 months (95% CI, 35.09–43.91) in the PA-LT group. Both the PA-TACE and PA-LT groups demonstrated significantly longer RFS than the LR group (both p < 0.001), while no significant difference was observed between the PA-TACE and PA-LT groups (p = 0.348). The 1-, 2-, 3-, and 4-year RFS rates were significantly higher in both the PA-TACE and PA-LT groups than in the LR group, while no significant differences were observed between the two groups at any time point (p = 0.294, 0.109, 0.283, and 0.146, respectively). However, the 5-year RFS rate was significantly higher in the PA-TACE group than in the PA-LT group (p = 0.005).

**Figure 2:**
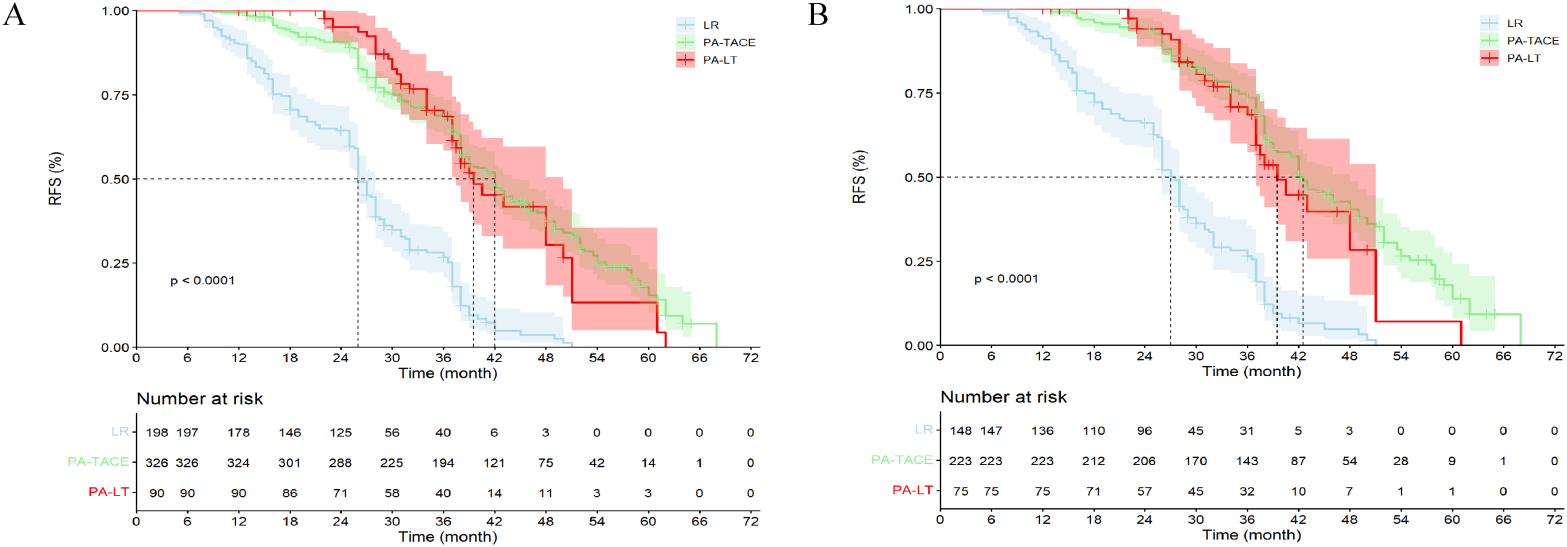
Kaplan–Meier curves for RFS in patients with HCC stratified by postoperative adjuvant treatment strategy. **(A)** the entire cohort; **(B)** the PSM cohort; RFS, recurrence-free survival; LR, liver resection; PA, postoperative adjuvant; TACE, transcatheter arterial chemoembolization; LT, lenvatinib plus tislelizumab.

In the PSM cohort, the median follow-up time was 46.00 months (95% CI, 44.83–47.17). Recurrence occurred in 122 patients (82.43%) in the LR group, 135 patients (60.54%) in the PA-TACE group, and 33 patients (44.00%) in the PA-LT group. mRFS was significantly longer in both the PA-TACE and PA-LT groups than in the LR group (both p < 0.001). Although the PA-TACE group demonstrated a numerically longer mRFS than the PA-LT group, the difference did not reach statistical significance (42.50 months (95% CI, 39.87–45.13) vs. 39.50 months (95% CI, 35.56–43.44), p = 0.150). Furthermore, the 1-, 2-, 3-, and 4-year RFS rates were significantly higher in both the PA-TACE and PA-LT groups compared with the LR group. Similarly, no significant differences in RFS rates were observed between the PA-TACE and PA-LT groups at any time point (p = 0.559, 0.581, 0.354, and 0.055 for the 1-, 2-, 3-, and 4-year RFS rates, respectively). Further details are shown in **Figure 2**; **Supplementary Table 2**.

### 3.3 Overall survival time

All deaths were attributed to tumor progression, recurrence-related liver failure, or deterioration in general condition. In the entire cohort, 74 patients (37.37%) in the LR group died, compared with 54 (16.56%) in the PA-TACE group and 17 (18.89%) in the PA-LT group. The median OS (mOS) was 52.00 months (95% CI, 48.91-55.09), 69.00 months (95% CI, 66.44-71.56), and 65.00 months (95% CI, 60.93-69.07), respectively (LR vs. PA-TACE and LR vs. PA-LT, both p < 0.001; PA-TACE vs. PA-LT, p = 0.572). Except for the absence of a statistically significant difference in 2-year OS rate between the LR and PA-LT groups (p = 0.436), both PA-TACE and PA-LT showed higher survival rates than LR at other evaluated time points. However, no significant differences in survival rates were observed between PA-TACE and PA-LT across all evaluated time point (2-, 3-, 4-, and 5-year OS rates: p = 0.329, 0.526, 0.718, and 0.196, respectively).

**Figure 3:**
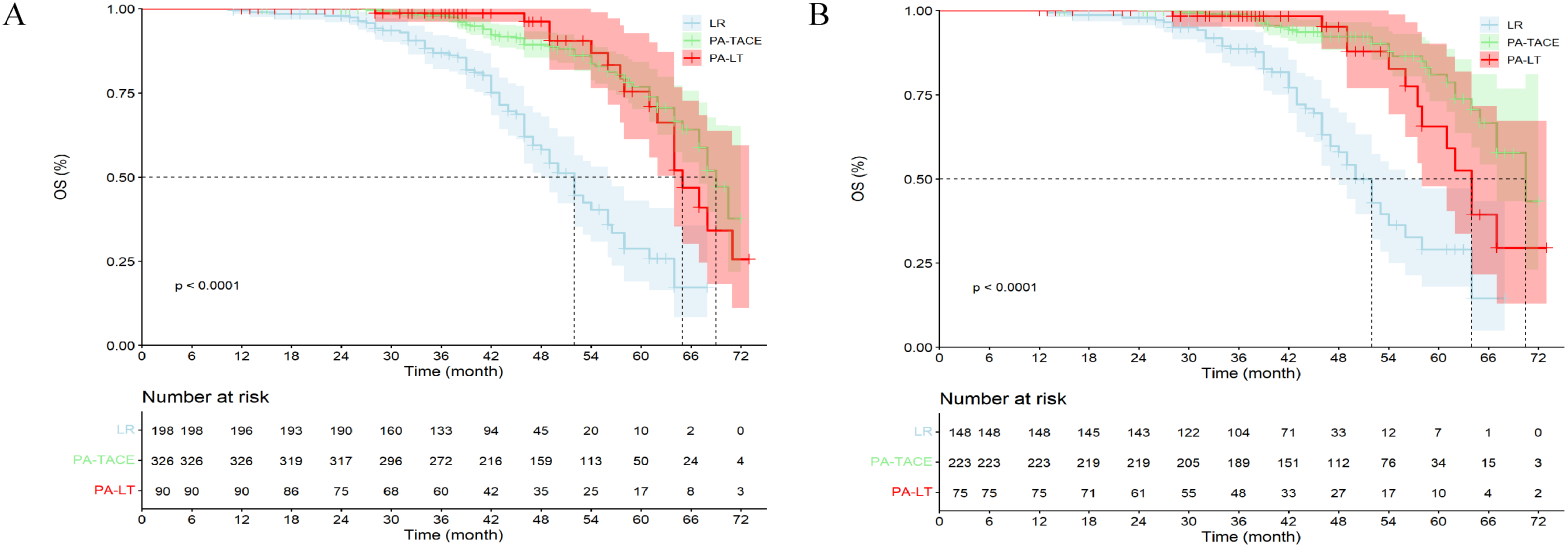
Kaplan–Meier curves for OS in patients with HCC stratified by postoperative adjuvant treatment strategy. **(A)** the entire cohort; **(B)** the PSM cohort; OS, overall survival; LR, liver resection; PA, postoperative adjuvant; TACE, transcatheter arterial chemoembolization; LT, lenvatinib plus tislelizumab.

In the PSM cohort, 53 patients (35.81%) in the LR group died, compared with 29 (13.00%) in the PA-TACE group and 13 (17.33%) in the PA-LT group. mOS was 52.00 months (95% CI, 48.67–55.33) in the LR group, 70.50 months (95% CI, 63.84–77.16) in the PA-TACE group, and 64.00 months (95% CI, 60.41–67.59) in the PA-LT group. Similar to the findings in the entire cohort, both the PA-TACE and PA-LT groups demonstrated significantly longer mOS than the LR group (both p < 0.001), whereas no statistically significant difference in mOS was observed between the PA-TACE and PA-LT groups (p = 0.080). However, given the relatively small number of events in both the PA-TACE and PA-LT groups across the entire and PSM cohorts, these findings should be interpreted with caution and require further validation in larger, prospective studies. Detailed results are presented in **Figure 3B** and **Supplementary Table 3**.

### 3.4 Cox regression analysis

Univariable and multivariable Cox regression analyses were performed in both the entire cohort and PSM cohort. In the entire cohort, multivariable analysis identified tumor diameter ≥ 5 cm (HR 2.892, 95% CI 2.311-3.618), multiple tumors (HR 3.015, 95% CI 2.194-4.393), ES grade 3/4 (HR 2.975, 95% CI 2.341-3.782), and the presence of MVI (HR 2.221, 95% CI 1.787-2.759) as independent risk factors for RFS. Compared with LR, both PA-TACE (HR 0.171, 95% CI 0.134-0.218) and PA-LT (HR 0.202, 95% CI 0.142-0.288) were independently associated with longer RFS. For OS, tumor diameter ≥ 5 cm (HR 2.152, 95% CI 1.467-3.158), BCLC stage B (HR 3.603, 95% CI 2.193-5.921), ES grade 3/4 (HR 3.162, 95% CI 2.198-4.547), and the presence of MVI (HR 2.992, 95% CI 1.991-4.498) were identified as independent risk factors. In contrast, both PA-TACE (HR 0.174, 95% CI 0.118-0.255) and PA-LT (HR 0.209, 95% CI 0.115-0.330) were independently associated with improved OS.

In the PSM cohort, tumor diameter ≥ 5 cm (HR 2.473, 95% CI 1.927-3.173), BCLC stage B (HR 3.237, 95% CI 2.146-4.882), ES grade 3/4 (HR 3.176, 95% CI 2.315-4.359), and the presence of MVI (HR 1.880, 95% CI 1.454-2.431) were identified as independent risk factors for RFS. In contrast, both PA-TACE (HR 0.178, 95% CI 0.134-0.236) and PA-LT (HR 0.214, 95% CI 0.144-0.320) were independently associated with improved RFS. For OS, tumor diameter ≥ 5 cm (HR 2.123, 95% CI 1.334–3.379), ES grade 3/4 (HR 4.067, 95% CI 2.465–6.709), and the presence of MVI (HR 2.897, 95% CI 1.735–4.775) were independent risk factors, whereas PA-TACE (HR 0.182, 95% CI 0.111–0.299), PA-LT (HR 0.268, 95% CI 0.140–0.514), and non-anatomical liver resection (HR 0.483, 95% CI 0.278–0.843) were independently associated with improved OS. Further details are provided in **Table 2** and **Supplementary Table 4**.

**Table 2A:** Multivariable Cox regression analysis of RFS in the entire cohort and PSM cohort.

Characteristics		The entire cohort		The PSM cohort	
		HR (95% CI)	P	HR (95% CI)	P
Type of treatment	LR	Reference		Reference	
	PA-TACE	0.171 (0.134, 0.218)	<0.001	0.178 (0.134, 0.236)	<0.001
	PA-LT	0.202 (0.142, 0.288)	<0.001	0.214 (0.144, 0.320)	<0.001
Gender (Male vs Female)		1.210 (0.890, 1.640)	0.223	–	–
AFP, ng/ml (≥ 200 vs < 200)		1.153 (0.935, 1.423)	0.183	–	–
Tumor diameter, cm (≥ 5 vs < 5)		2.892 (2.311, 3.618)	<0.001	2.473 (1.927, 3.173)	<0.001
Tumor number (Multiple vs Single)		3.015 (2.194, 4.393)	<0.001	–	–
BCLC stage (B vs 0+A)		1.413 (0.924, 2.161)	0.111	3.237 (2.146, 4.882)	<0.001
ES (3/4 vs 1/2)		2.975 (2.341, 3.782)	<0.001	3.176 (2.315, 4.359)	<0.001
MVI (Positive vs Negative)		2.221 (1.787, 2.759)	<0.001	1.880 (1.454, 2.431)	<0.001
ALBI grade (2 vs 1)		1.019 (0.792, 1.311)	0.885	–	–
Child-Pugh score (6 vs 5)		0.993 (0.714, 1.380)	0.964	–	–
ALP, U/L		1.001 (1.000, 1.001)	0.424	1.002 (0.999, 1.005)	0.211
γ-GGT, U/L		1.001 (1.000, 1.001)	0.154	1.001 (1.000, 1.002)	0.166

PSM, propensity score matching; LR, liver resection; PA, postoperative adjuvant; TACE, transcatheter arterial chemoembolization; LT, lenvatinib plus tislelizumab; AFP, alpha-fetoprotein; BCLC, Barcelona Clinic Liver Cancer; ES, Edmondson-Steiner; MVI, microvascular invasion; ALBI, albumin-bilirubin; ALP, alkaline phosphatase; γ-GGT, gamma-glutamyl transferase.

**Table 2B:** Multivariable Cox regression analysis of OS in the entire cohort and PSM cohort.

Characteristics		The entire cohort		The PSM cohort	
		HR (95% CI)	P	HR (95% CI)	P
Type of treatment	LR	Reference		Reference	
	PA-TACE	0.174 (0.118, 0.255)	<0.001	0.182 (0.111, 0.299)	<0.001
	PA-LT	0.209 (0.115, 0.330)	<0.001	0.268 (0.140, 0.514)	<0.001
Tumor diameter, cm (≥ 5 vs < 5)		2.152 (1.467, 3.158)	<0.001	2.123 (1.334, 3.379)	0.001
AFP, ng/ml (≥ 200 vs < 200)		1.211 (0.850, 1.725)	0.290	1.074 (0.700, 1.648)	0.743
BCLC (B vs 0+A)		3.603 (2.193, 5.921)	<0.001	–	–
ES (3/4 vs 1/2)		3.162 (2.198, 4.547)	<0.001	4.067 (2.465, 6.709)	<0.001
MVI (Positive vs Negative)		2.992 (1.991, 4.498)	<0.001	2.897 (1.735, 4.775)	<0.001
Resection pattern (Nonanatomic vs Anatomic)		0.764 (0.499, 1.004)	0.270	0.483 (0.278, 0.843)	0.010
Platelet, 10^9^/L		1.001 (0.999, 1.004)	0.270		
ALP, U/L		1.003 (0.999, 1.006)	0.067		
γ-GGT, U/L		1.000 (0.999, 1.001)	0.749	1.000 (0.998, 1.003)	0.719
Hemoglobin, g/L		–	–	1.002 (0.998, 1.005)	0.394
Total protein, g/L		–	–	1.000 (0.998, 1.003)	0.719

PSM, propensity score matching; LR, liver resection; PA, postoperative adjuvant; TACE, transcatheter arterial chemoembolization; LT, lenvatinib plus tislelizumab; AFP, alpha-fetoprotein; BCLC, Barcelona Clinic Liver Cancer; ES, Edmondson-Steiner; MVI, microvascular invasion; ALBI, albumin-bilirubin; ALBI grade 1, ≤ -2.60; ALP, alkaline phosphatase; γ-GGT, gamma-glutamyl transferase.

### 3.5 Inverse probability of treatment weighting

Stabilized IPTW weights were constructed based on propensity scores estimated using a multinomial logistic regression model, and 1st–99th percentile truncation was applied (final weight range: 0.4766–1.9558) to reduce the influence of extreme weights. After weighting, the effective sample sizes of the LR, PA-TACE, and PA-LT groups were 182.5, 312.5, and 74.7, respectively. Covariate balance was assessed using SMDs. Except for resection pattern (SMD = 0.142), all remaining covariates achieved good balance with SMDs < 0.10, and the SMD for resection pattern was substantially reduced after weighting. A comparison of SMDs before and after weighting is shown in **Supplementary Figure 1**.

**Figure 4:**
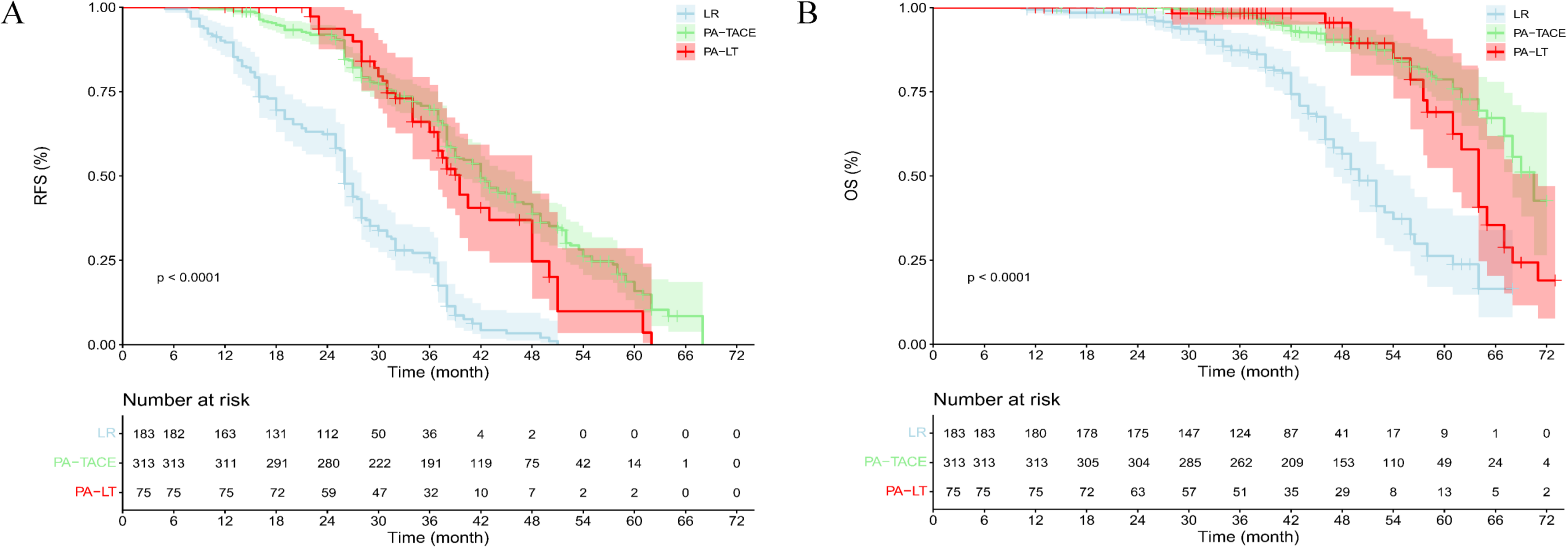
IPTW-adjusted Kaplan–Meier curves. **(A)** the recurrence-free survival; **(B)** the overall survival; LR, liver resection; PA, Postoperative adjuvant; TACE, transcatheter arterial chemoembolization; LT, lenvatinib plus tislelizumab.

IPTW-adjusted Cox regression analysis showed that, compared with the LR group, both the PA-TACE and PA-LT groups had significantly lower risks of recurrence (weighted HRs: 0.223, 95% CI 0.178-0.280, p < 0.001; and 0.295, 95% CI 0.217-0.402, p < 0.001, respectively). Similarly, both groups demonstrated significantly reduced risks of death (weighted HRs: 0.190, 95% CI 0.125-0.289, p < 0.001; and 0.338, 95% CI 0.210-0.543, p < 0.001, respectively). However, no significant differences in RFS or OS were observed between the PA-TACE and PA-LT groups (RFS: weighted HR 0.756, 95% CI 0.516-1.108, p = 0.145; OS: weighted HR 0.562, 95% CI 0.298-1.059, p = 0.076). The weighted Kaplan-Meier curves further demonstrated clear separation between each adjuvant treatment group and the LR group, whereas no obvious separation was observed between the two adjuvant treatment groups (**Figure 4**). These findings were consistent with the PSM analysis, further supporting the robustness of the study findings.

### 3.6 Subgroup analysis

**Figure 5:**
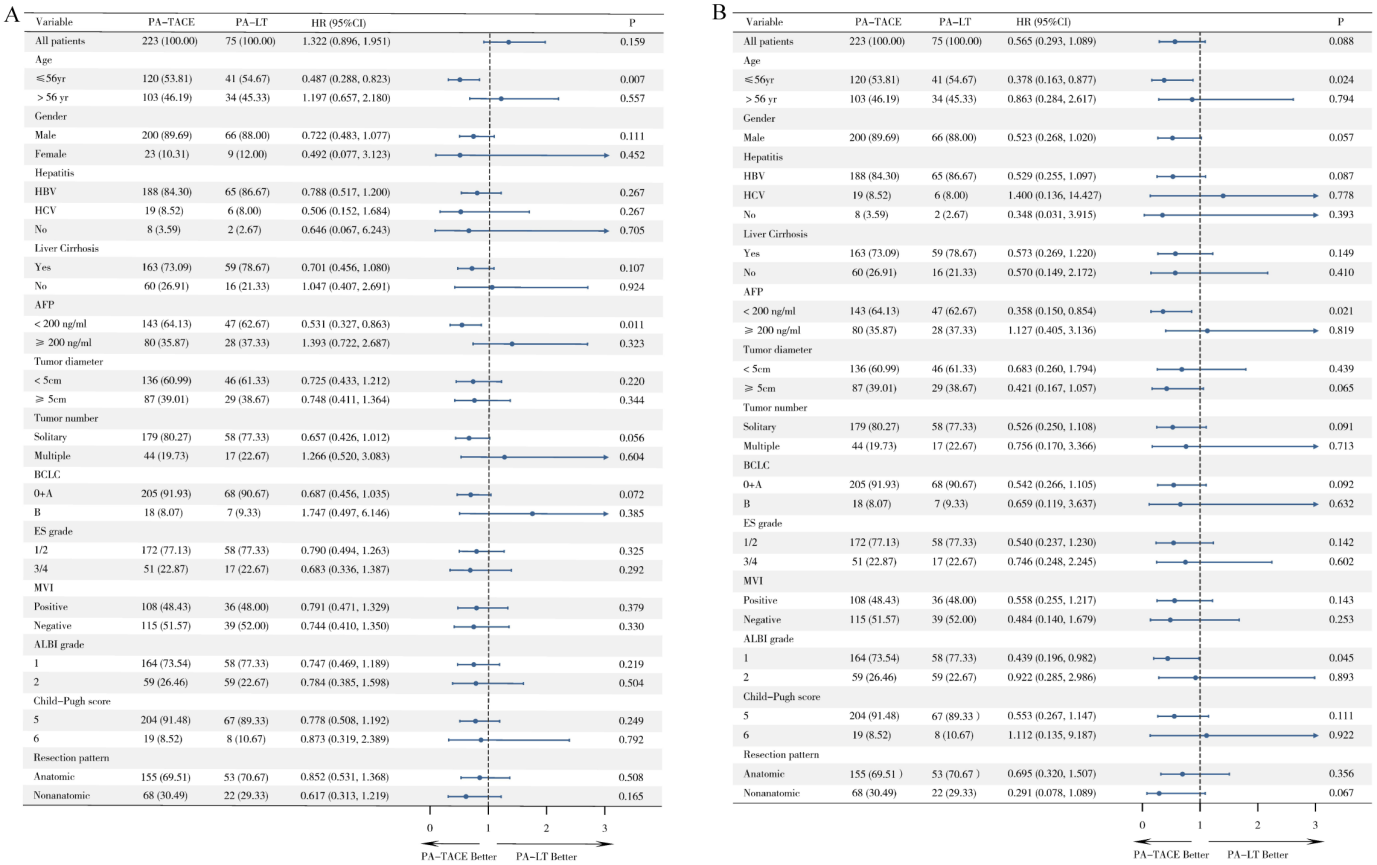
Forest plots for subgroup analyses of RFS and OS comparing PA-LT with PA-TACE in patients with HCC. **(A)** recurrence-free survival (RFS); **(B)** overall survival (OS); PA, postoperative adjuvant; TACE, transcatheter arterial chemoembolization; LT, lenvatinib plus tislelizumab; HBV, hepatitis B virus; HCV, hepatitis C virus; AFP, alpha-fetoprotein; BCLC, Barcelona Clinic Liver Cancer; ES, Edmondson-Steiner; MVI, microvascular invasion; ALBI, albumin-bilirubin.

To further explore the potential value of PA-TACE and PA-LT in improving RFS and OS, subgroup analyses were performed. In the PSM cohort, compared with the PA-LT group, the PA-TACE group was associated with improved RFS among patients aged ≤ 56 years (HR 0.487, 95% CI 0.288–0.823) and those with serum AFP ≤ 200 ng/ml (HR 0.531, 95% CI 0.327–0.863). Similarly, the PA-TACE group was associated with better OS than the PA-LT group among patients aged ≤ 56 years (HR 0.378, 95% CI 0.163–0.877) and those with serum AFP ≤ 200 ng/mL (HR 0.358, 95% CI 0.150–0.854). No significant differences in RFS or OS were observed between the PA-TACE and PA-LT groups in the remaining subgroups. Detailed results are presented in **Figure 5**.

### 3.7 Efficacy of the number of PA-TACE treatments on patients’ outcomes

Patients were stratified into groups with one, two, or three to four HRRFs, including tumor size ≥ 5 cm, multiple tumors, presence of MVI, and ES grade 3/4. We further evaluated the efficacy of different numbers of PA-TACE sessions across different recurrence risk burdens. The results showed that, among patients with three to four HRRFs, those who received 4–5 PA-TACE sessions achieved a longer mRFS than those who received 1–2 PA-TACE sessions. In contrast, among patients with one or two HRRFs, although differences in mRFS were observed among patients receiving different numbers of PA-TACE sessions, no statistically significant differences were observed overall between the groups (see **Table 3** for details). Due to the limited number of death events across all subgroups, the statistical power for subgroup analyses of OS was insufficient; therefore, no subgroup analyses of OS were performed in this study.

**Table 3:** Association between the number of PA-TACE treatments and RFS stratified by HRRFs.

Number	mRFS (95%CI), months	P				
		1 session	2 sessions	3 sessions	4 sessions	5 sessions
One HRRF						
1 session	50.00 (35.52, 64.48)	–	0.962	0.086	0.102	0.094
2 sessions	42.00 (38.29, 45.71)	0.962	–	0.056	0.052	0.077
3 sessions	52.00 (47.62, 56.39)	0.086	0.056	–	0.982	0.819
4 sessions	50.00 (43.94, 56.06)	0.102	0.052	0.982	–	0.777
5 sessions	52.00 (46.08, 57.92)	0.094	0.077	0.819	0.777	–
Two HRRFs						
1 session	31.00 (20.36, 41.65)		0.933	0.155	0.189	0.091
2 sessions	34.00 (29.00, 39.00)	0.993		0.149	0.120	0.051
3 sessions	38.00 (32.68, 43.23)	0.155	0.149		0.607	0.250
4 sessions	46.00 (33.88, 58.12)	0.189	0.120	0.607		0.524
5 sessions	39.00 (27.52, 50.48)	0.091	0.051	0.250	0.524	
Three to four HRRFs						
1 session	18.00 (14.15, 21.85)		0.430	0.275	0.033	0.025
2 sessions	25.00 (22.13, 28.87)	0.430		0.407	0.005	0.005
3 sessions	26.00 (21.36, 30.64)	0.275	0.407		0.096	0.146
4 sessions	28.00 (NA, NA)	0.033	0.005	0.096		0.317
5 sessions	28.00 (NA, NA)	0.025	0.005	0.146	0.317	

mRFS, median recurrence-free survival; HRRFs, high-risk recurrence factors, included tumor size ≥ 5 cm, multiple tumors, presence of MVI, and ES grade 3/4.

### 3.8 Efficacy of PA-LT duration on patients’ survival outcomes

**Figure 6:**
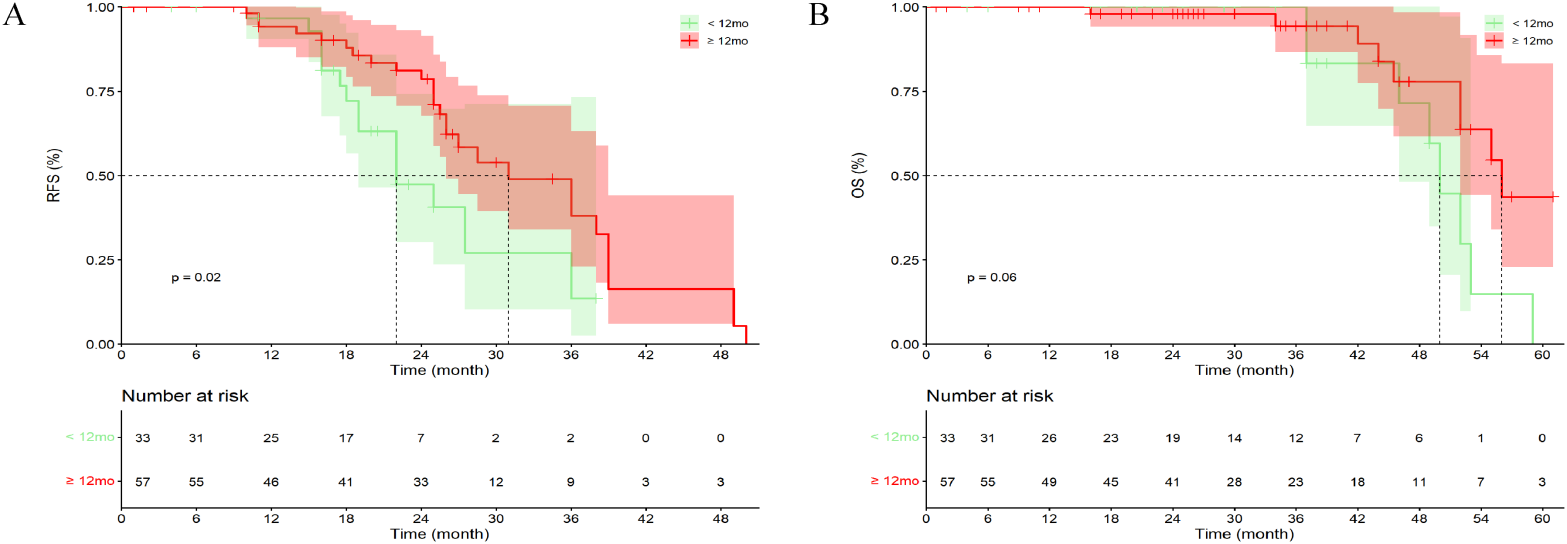
Twelve-month landmark analysis of RFS and OS according to PA-LT duration. RFS, recurrence-free survival; OS, OS, overall survival; PA, postoperative adjuvant; LT, lenvatinib plus tislelizumab; Landmark mRFS (95%CI): < 12 months, 22.00 months (16.35–27.64); ≥ 12 months, 31.00 months (23.05–38.95); Landmark mOS (95%CI): < 12 months, 50.00 months (47.67–52.33); ≥ 12 months, 56.00 months (50.37–61.63).

Among patients who received PA-LT in the entire cohort, all had a treatment duration of at least 6 months. According to Chen et al (25), a 12-month cutoff was used for subgroup stratification based on PA-LT duration. To minimize potential guarantee-time bias associated with treatment duration, a 12-month landmark analysis was subsequently performed. Patients were stratified into two groups: 33 patients (36.67%) received PA-LT for < 12 months, whereas 57 patients (63.33%) received PA-LT for ≥ 12 months. In the landmark analysis, patients receiving PA-LT for ≥ 12 months demonstrated significantly longer mRFS than those treated for < 12 months (HR 0.464, 95% CI 0.237–0.911, p = 0.020). However, no statistically significant difference in OS was observed between the two groups (HR 0.410, 95% CI 0.156–1.082, p = 0.060). Further details are provided in **Figure 6**.

### 3.9 Adverse events in the PA-TACE and PA-LT groups

Most AEs in the PA-TACE and PA-LT groups were mild and were managed symptomatically during hospitalization. A small proportion of patients experienced CTCAE grade 3 treatment-related AEs. No CTCAE grade 4 or 5 treatment-related AEs were observed. The most common AEs in the PA-TACE group were pain (40.49%), elevated transaminases (38.04%), nausea/vomiting (31.29%), fever (29.19%), and thrombocytopenia (27.61%). In the PA-LT group, the most frequent AEs were hypertension (27.78%), elevated transaminases (22.22%), neutropenia (20.00%), thrombocytopenia (17.78%), and fatigue (17.78%). Detailed AE data are provided in **Supplementary Table 5**.

## 4 Discussion

Radical liver resection remains the cornerstone of treatment for HCC and offers the potential for long-term survival (26). Nonetheless, postoperative recurrence continues to pose a major clinical challenge, substantially compromising long-term outcomes. The presence of multiple tumors, tumor diameter ≥ 5 cm, poor differentiation, and vascular invasion are associated with poorer prognosis after radical liver resection (6–8). Given the limited therapeutic options available after recurrence, prevention of postoperative recurrence is essential. Although numerous studies have investigated the comparative efficacy of different postoperative adjuvant therapies, the existing literature remains incomplete (27–29). In this study, we compared the effectiveness of PA-TACE and PA-LT in patients who underwent radical liver resection to provide real-world evidence for postoperative treatment selection in this area of clinical uncertainty.

This study demonstrates that both PA-TACE and PA-LT were associated with longer RFS and OS in HCC patients. Wang et al. reported that patients with intermediate (tumor size > 5 cm) or high risk of recurrence (including single tumor with microvascular invasion or 2–3 tumors) may benefit from PA-TACE, with improved 3-year OS compared with liver resection alone (85.20% vs. 77.40%; p = 0.040) (30). A multicenter retrospective study showed that, among patients with MVI after LR, PA-TACE was associated with significantly improved RFS and OS compared with LR (RFS: HR 0.693, 95% CI 0.523–0.918, p = 0.009; OS: HR 0.628, 95% CI 0.453–0.871, p = 0.023) (12). In another multicenter subgroup analysis, PA-TACE was likewise shown to significantly improve mRFS in CNLC stage I–II HCC patients with MVI after radical resection compared with LR (CNLC I, 37.00 vs 15.00 months, p < 0.001; CNLC II, 25.00 vs 11.00 months, p = 0.048), as well as mOS (CNLC I, not reached vs 32.00 months p < 0.001; CNLC II, not reached vs 26.00 months, p = 0.002). However, no improvement in mRFS or mOS was observed in patients without MVI (CNLC stage I, mRFS, p = 0.362, mOS, p = 0.841; CNLC stage II, mRFS, p = 0.697, mOS, p = 0.087, respectively) (13). Consistent findings were observed in our study, where PA-TACE was associated with longer RFS and OS compared with LR (RFS: HR 0.178, 95% CI 0.134-0.236, p < 0.001; OS: HR 0.182, 95% CI 0.111–0.299, p < 0.001). In studies investigating adjuvant targeted therapy and immunotherapy strategies, the IMbrave050 trial represented a breakthrough by demonstrating the efficacy of atezolizumab plus bevacizumab as an adjuvant regimen following resection or ablation (31). Similarly, subgroup analyses from another retrospective study showed that postoperative adjuvant tyrosine kinase inhibitors combined with anti-PD-1 antibodies significantly improved RFS in patients with multiple tumors (HR 0.300, 95% CI 0.090–0.980, p = 0.046), tumor diameter > 5 cm (HR 0.450, 95% CI 0.240–0.860, p = 0.015), and vascular invasion (HR 0.360, 95% CI 0.170–0.760, p = 0.007) (32). Another study demonstrated that, compared with LR, PA-lenvatinib combined with PD-1 blockade significantly reduced the 3-year RFS risk in patients with MVI (HR 0.591, 95% CI 0.395-0.885, p = 0.012), although no significant improvement in 3-year OS was observed (HR 0.764, 95% CI 0.411-1.423, p = 0.390) (33). In our previous study, we found that compared with LR alone, PA-LT was associated with significantly prolonged mRFS (HR 0.510,95% CI 0.280-0.910, p = 0.021) and mOS (HR 0.360, 95% CI 0.180-0.700, p = 0.002) (22). In this study, both mRFS and mOS were significantly longer in the PA-LT group than in the LR group (both p < 0.001). These findings are broadly consistent with those reported in previous studies.

TACE targets tumor cells released during surgical manipulation, as well as microscopic lesions undetectable by imaging or intraoperative assessment (34). By administering iodized oil via the arteries supplying the tumor, TACE reduces blood flow within the tumor region, thereby depriving the tumor of oxygen and nutrients. Direct intratumoral delivery of chemotherapeutic agents maximizes their concentration within tumor tissue, thereby effectively killing tumor cells. Meanwhile, iodized oil prolongs local drug retention, enhancing exposure to chemotherapy and thereby inhibiting tumor recurrence (35, 36). However, TACE-induced vascular embolization can trigger ischemic injury, upregulate hypoxia-inducible factor-1α and vascular endothelial growth factor, thereby exacerbating hypoxia and immune suppression within the tumor microenvironment, which may paradoxically facilitate tumor progression (37). Several studies have shown no additional benefit of multiple PA-TACE sessions compared to a single session (12, 38). Therefore, for HCC patients with HRRFs, 1–2 cycles of PA-TACE therapy are routinely recommended (39). In our study, we similarly observed that among patients with one or two HRRFs, no significant differences in outcomes were found across those who received 1–5 sessions of PA-TACE. In contrast, among patients with three to four HRRFs, those who received 4–5 sessions of PA-TACE achieved a longer mRFS than those who received 1–2 sessions. Therefore, whether increasing the number of PA-TACE sessions can further enhance the adjuvant therapeutic effect in patients with a greater high-risk burden of HCC remains to be further investigated.

In contrast, the combination of TKIs and PD-1 inhibitors exerts synergistic antitumor effects through vascular normalization and immune microenvironment modulation. In patients with HCC, the immune microenvironment manifests a pronounced abundance of immune cell infiltration, encompassing T cells, natural killer cells, macrophages, and dendritic cells (40, 41). PD-1 inhibitors enhance antitumor immunity by activating CD8^+^ T cells, increasing the phagocytic activity of tumor-associated macrophages (TAMs), and reducing TAM M2 polarization to eliminate tumor cells (15, 42). TKIs enhance dendritic cell activation, promote T-cell infiltration, improve antigen presentation, and activate interferon signaling. In addition, they can transiently normalize tumor angiogenesis, improve the tumor microenvironment, and enhance the responsiveness of tumor cells to PD-1 inhibitors (43–45). Therefore, the combination of tislelizumab and lenvatinib may eradicate occult microscopic malignant lesions in the liver, thereby effectively reducing tumor recurrence (46).

In this study, although patients in the PA-TACE group exhibited longer RFS than those in the PA-LT group, the intergroup difference did not reach statistical significance, suggesting that the two treatment strategies may achieve comparable anti-recurrence effects through distinct mechanisms of action. Subgroup analysis showed that, except for patients aged ≤ 56 years and those with serum AFP ≤ 200 ng/ml, all other subgroups showed trends consistent with the overall findings. However, because the overall sample size of this study was relatively small, further stratification resulted in even smaller subgroup sizes, which may have affected the statistical power and robustness of the results. Therefore, the potential differences in benefit between these two treatment approaches across different patient subpopulations still require further validation in large-scale studies.

The ideal duration of PA-LT therapy remains unclear. Su et al. conducted a prospective multicenter cohort study and demonstrated that patients who received adjuvant immunotherapy for more than six months had longer RFS and OS than those treated for six months or less (RFS: HR 0.660, 95% CI 0.420–1.040, p = 0.071; OS: HR 0.590, 95% CI 0.300–1.170, p = 0.128). Although the differences were not statistically significant, these findings still suggest that a treatment duration of six months may still be insufficient (16). Furthermore, subgroup analysis by Chen et al. showed that patients who received adjuvant immunotherapy for 12 months or longer achieved significantly improved RFS compared with those treated for less than 12 months (HR 0.460, 95% CI 0.210-0.990, p = 0.041), without a significant increase in adverse events (25). Consistent with these findings, we likewise observed that patients who received PA-LT for 12 months or longer achieved significantly longer RFS than those treated for less than 12 months (HR 0.464, 95% CI 0.237–0.911, p = 0.020). However, given the limited available evidence and the potential influence of inter-patient heterogeneity, the appropriate duration of targeted immunotherapy remains to be determined and warrant further prospective investigation.

Several limitations warrant consideration. First, although PSM was applied to reduce confounding, the retrospective design inherently limits causal inference, and residual selection bias cannot be excluded, which may have influenced baseline tumor characteristics and outcomes. Second, the relatively small sample size, particularly in the PA-LT subgroup, may limit statistical power and increase the risk of type II error. Third, the predominance of HBV-related HCC may limit the generalizability of our findings to HCC caused by other etiologies. Furthermore, incomplete HBV DNA data prevented evaluation of the potential impact of viral load on recurrence risk. Finally, because a considerable proportion of patients had not reached the OS endpoint at the time of analysis, longer follow-up is required for more definitive results.

## 5 Conclusion

Both PA-TACE and PA-LT were associated with favorable survival outcomes in HCC patients with HRRFs after radical resection, with acceptable safety profiles. However, no significant differences were observed between the two adjuvant treatment strategies.

## Data Availability

The raw data supporting the conclusions of this article will be made available by the authors, without undue reservation.
