# Supplementary material for: Comparative efficacy of postoperative adjuvant transcatheter arterial chemoembolization versus lenvatinib plus tislelizumab in patients with BCLC stage 0 -B hepatocellular carcinoma after radical resection
Source: Front Immunol. 2026 Jun 29;17:1778330. doi: 10.3389/fimmu.2026.1778330 (PMC13358005; doi:10.3389/fimmu.2026.1778330)
Supplement: Supplementary file 1 [file Table1.docx]

**Comparative Efficacy of Postoperative Adjuvant Transcatheter Arterial Chemoembolization versus Lenvatinib Plus Tislelizumab in Patients with BCLC stage 0 -B**

Xu Feng^1,2+^, Yupei Ao^3+^, Lili Wang^1^, Kai Chen^2^, Chengjia Tang^1,2^, and Chengcheng Huang^4*^

^1^ Department of Hepatobiliary Surgery, The First Affiliated Hospital of Chongqing Medical University, (China), Chongqing

^2^ Department of Hepatobiliary Surgery, The Affiliated Yongchuan Hospital of Chongqing Medical University, (China), Chongqing

^3^ Health Screening Centre, Chongqing Western Hospital, (China), Chongqing

^4^ Department of Geriatrics, The Affiliated Yongchuan Hospital of Chongqing Medical University, (China), Chongqing

^+^ Xu Feng and Yupei Ao have contributed equally to this work.

^*^ Correspondence should be addressed to Chengcheng Huang; 854899724@qq.com.

Supplementary table 1 Detailed information on all drugs used in this study

| Drug | manufacturer, company name, and country of origin |
| --- | --- |
| Epirubicin hydrochloride | Pharmorubicin, Pfizer (Wuxi) Co., Ltd., Pfizer Inc., Wuxi, Jiangsu, China |
|  | Zhejiang Hisun Pharmaceutical Co., Ltd., Zhejiang Hisun Pharmaceutical Co., Ltd., Hangzhou, Zhejiang, China |
|  | Aidashan®, Hanhui Pharmaceutical Co., Ltd., Hanhui Pharmaceutical Co., Ltd., Hangzhou, Zhejiang, China |
| Pirarubicin hydrochloride | Therarubicin®, Meiji Seika Kaisha, Ltd., Tokyo, Japan |
|  | Shenzhen Main Luck Pharmaceuticals Inc., Shenzhen, Guangdong, China |
|  | Zhejiang Hisun Pharmaceutical Co., Ltd., Hangzhou, Zhejiang, China |
| Doxorubicin hydrochloride | Adriamycin, Pfizer Inc., New York, NY, USA |
|  | Aidashan®, Hanhui Pharmaceutical Co., Ltd., Hangzhou, Zhejiang, China |
|  | Zhejiang Hisun Pharmaceutical Co., Ltd., Hangzhou, Zhejiang, China |
|  | Shenzhen Main Luck Pharmaceuticals Inc., Shenzhen, Guangdong, China |
| Oxaliplatin | Jiangsu Hengrui Pharmaceuticals Co., Ltd., Lianyungang, Jiangsu, China |
|  | Zhejiang Hisun Pharmaceutical Co., Ltd., Hangzhou, Zhejiang, China |
|  | Qi'ao®, Qilu Pharmaceutical (Hainan) Co., Ltd., Qilu Pharmaceutical Group, Haikou, Hainan, China |
| Fluorouracil | Qilu Pharmaceutical Co., Ltd., Jinan, Shandong, China |
|  | Zhejiang Hisun Pharmaceutical Co., Ltd., Hangzhou, Zhejiang, China |
|  | Shanghai Xudong Haipu Pharmaceutical Co., Ltd., Shanghai, China |
| lipiodol | Shanghai Xudong Haipu Pharmaceutical Co., Ltd., Shanghai, China |
|  | Yantai Luyin Pharmaceutical Co., Ltd., Yantai, Shandong, China |
|  | Qi'ao®, Qilu Pharmaceutical (Hainan) Co., Ltd., Qilu Pharmaceutical Group, Haikou, Hainan, China |
| Lenvatinib | LENVIMA®; Eisai, Japan |
|  | Zewanxin®; Nanjing Chia-Tai Tianqing, China |
| Tislelizumab | Baize'an®; Boehringer Ingelheim Biopharma (China), BeiGene, Ltd., China |

Supplementary table 2 Comparison of RFS in HCC patients stratified by different postoperative adjuvant therapy strategies

| Characteristics | | mRFS (months) | 1-year RFS rate (%) | 2-year RFS rate (%) | 3-year RFS rate (%) | 4-year RFS rate (%) | 5-year RFS rate (%) |
| --- | --- | --- | --- | --- | --- | --- | --- |
| the Entire cohort | LR | 26.00 (25.03-26.97) | 89.90 (85.78-94.02) | 64.40 (61.14-71.06) | 26.70 (20.04-33.36) | 2.50 (0-5.64) | - |
|  | PA-TACE | 42.00 (39.76-4.24) | 98.50 (97.13-99.87) | 90.30 (87.16-93.44) | 67.80 (62.51-73.09) | 38.20 (32.12-44.28) | 15.90 (9.63-22.17) |
|  | PA-LT | 39.50 (35.09-43.91) | 100.00 (100.00-100.00) | 95.10 (90.40-99.80) | 68.60 (56.64-80.56) | 29.90 (16.61-43.19) | 4.40 (0-12.63) |
| the PSM cohort | LR | 27.00 (26.00-28.00) | 91.20 (86.68-95.71) | 66.10 (61.59-70.61) | 26.50 (18.66-34.34) | 3.30 (0-7.42) | - |
|  | PA-TACE | 42.50 (39.87-45.13) | 99.10 (97.92-100.00) | 92.70 (89.17-96.23) | 73.60 (67.52-79.68) | 40.40 (34.32-46.78) | 13.90 (6.26-21.54) |
|  | PA-LT | 39.50 (35.56-43.44) | 100.00 (100.00-100.00) | 94.10 (88.42-99.78) | 68.60 (56.25-80.95) | 28.40 (10.17-46.63) | - |
| Comparison | | p value | | | | | |
| the Entire cohort | LR vs PA-TACE | <0.001 | <0.001 | <0.001 | <0.001 | <0.001 | - |
|  | LR vs PA-LT | <0.001 | 0.002 | <0.001 | <0.001 | <0.001 | - |
|  | PA-TACE vs PA-LT | 0.348 | 0.294 | 0.109 | 0.283 | 0.146 | 0.005 |
| the PSM cohort | LR vs PA-TACE | <0.001 | <0.001 | <0.001 | <0.001 | <0.001 | - |
|  | LR vs PA-LT | <0.001 | 0.019 | <0.001 | <0.001 | <0.001 | - |
|  | PA-TACE vs PA-LT | 0.150 | 0.559 | 0.581 | 0.354 | 0.055 | - |

PSM, propensity score matching; mRFS, median recurrence-free survival time; LR, liver resection; PA, postoperative adjuvant; TACE, transcatheter arterial chemoembolization; LT, lenvatinib plus tislelizumab.

Supplementary table 3 Comparison of OS in HCC patients stratified by different postoperative adjuvant therapy strategies

| Characteristics | | mOS (months) | 2-year OS rate (%) | 3-year OS rate (%) | 4-year OS rate (%) | 5-year OS rate (%) |
| --- | --- | --- | --- | --- | --- | --- |
| the Entire cohort | LR | 52.00 (48.91-55.09) | 97.40 (95.24-99.56) | 86.90 (81.80-92.00) | 58.10 (49.08-67.12) | 25.80 (14.04-37.56) |
|  | PA-TACE | 69.00 (66.44-71.56) | 99.70 (99.11-100.00) | 97.60 (95.84-99.36) | 88.70 (84.58-92.82) | 76.90 (70.04-83.76) |
|  | PA-LT | 65.00 (60.93-69.07) | 98.70 (96.15-100.00) | 98.70 (96.15-100.00) | 90.50 (81.29-99.71) | 70.90 (54.04-87.76) |
| the PSM cohort | LR | 52.00 (48.67-55.33) | 98.00 (95.65-100.00) | 88.60 (83.11-94.09) | 57.90 (47.12-68.68) | 29.00 (14.88-43.11) |
|  | PA-TACE | 70.50 (63.84–77.16) | 100.00 (100.00-100.00) | 98.50 (96.74-100.00) | 92.20 (88.08, 96.32) | 78.70 (69.29-88.11) |
|  | PA-LT | 64.00 (60.41–67.59) | 100.00 (100.00-100.00) | 100.00 (100.00-100.00) | 87.90 (76.34-99.46) | 65.60 (44.63-86.57) |
| Comparison | | p value | | | | |
| the Entire cohort | LR vs PA-TACE | <0.001 | 0.021 | <0.001 | <0.001 | <0.001 |
|  | LR vs PA-LT | <0.001 | 0.436 | 0.001 | <0.001 | <0.001 |
|  | PA-TACE vs PA-LT | 0.572 | 0.329 | 0.526 | 0.718 | 0.196 |
| the PSM cohort | LR vs PA-TACE | <0.001 | 0.063 | <0.001 | <0.001 | <0.001 |
|  | LR vs PA-LT | <0.001 | 0.290 | 0.002 | <0.001 | <0.001 |
|  | PA-TACE vs PA-LT | 0.080 | 1.000 | 0.418 | 0.245 | 0.018 |

PSM, propensity score matching; mOS, median Overall survival; LR, liver resection; PA, postoperative adjuvant; TACE, transcatheter arterial chemoembolization; LT, lenvatinib plus tislelizumab.

Supplementary table 4.A Univariate Cox regression analysis of RFS in the entire cohort and PSM cohort

| Characteristics | | the Entire cohort | | the PSM cohort | |
| --- | --- | --- | --- | --- | --- |
|  |  | HR (95% CI) | P | HR (95% CI) | P |
| Type of treatment | LR | Reference | - | Reference | - |
|  | PA-TACE | 0.258 (0.206, 0.323) | <0.001 | 0.224 (0.171, 0.294) | <0.001 |
|  | PA-LT | 0.278 (0.198, 0.391) | <0.001 | 0.288 (0.196, 0.425) | <0.001 |
| Age, yr (≤56 vs >56) | | 0.923 (0.760, 1.120) | 0.416 | 0.917 (0.727, 1.156) | 0.464 |
| Gender (Male vs Female) | | 1.352 (1.001, 1.828) | 0.049 | 1.216 (0.823, 1.798) | 0.326 |
| Hepatitis | No | Reference | - | Reference | - |
|  | HBV | 0.966 (0.696, 1.339) | 0.834 | 1.043 (0.551, 1.976) | 0.897 |
|  | HCV | 1.166 (0.728, 1.869) | 0.522 | 1.124 (0.527, 2.399) | 0.762 |
|  | AH | 1.056 (0.606, 1.839) | 0.848 | 0.752 (0.285, 1.982) | 0.564 |
| Liver Cirrhosis (Positive vs Negative) | | 1.010 (0.818, 1.247) | 0.927 | 0.918 (0.702, 1.199) | 0.528 |
| AFP, ng/ml (≥ 200 vs < 200) | | 1.329 (1.093, 1.640) | 0.005 | 1.269 (0.993, 1.621) | 0.057 |
| Tumor diameter, cm (≥ 5 vs < 5) | | 1.744 (1.437, 2.117) | <0.001 | 1.743 (1.382, 2.197) | <0.001 |
| Tumor number (Multiple vs Single) | | 1.417 (1.121, 1.791) | 0.004 | 1.193 (0.899, 1.584) | 0.222 |
| BCLC grade (B vs 0+A) | | 2.038 (1.488, 2.792) | <0.001 | 1.712 (1.156, 2.561) | 0.007 |
| ES (3/4 vs 1/2） | | 1.409 (1.128, 1.759) | 0.002 | 1.366 (1.039, 1.796) | 0.026 |
| MVI (Positive vs Negative) | | 1.406 (1.153, 1.714) | <0.001 | 1.342 (1.063, 1.694) | 0.013 |
| ALBI grade (2 vs 1) | | 1.295 (1.055, 1.591) | 0.014 | 1.214 (0.938, 1.570) | 0.140 |
| Child-pugh score (6 vs 5) | | 1.449 (1.105, 1.900) | 0.007 | 1.395 (0.977, 1.990) | 0.067 |
| Resection pattern (Nonanatomic vs Anatomic) | | 0.839 (0.675, 1.043) | 0.114 | 0.954 (0.739, 1.231) | 0.715 |
| Blood transfusion (Yes vs No) | | 1.256 (0.967, 1.632) | 0.088 | 1.157 (0.919, 1.458) | 0.215 |
| Blood loss, ml | | 1.000 (1.000, 1.000) | 0.218 | 1.000 (1.000, 1.000) | 0.458 |
| Operative time, min | | 1.000 (1.000, 1.000) | 0.448 | 1.020 (0.713, 1.459) | 0.914 |
| Hemoglobin, g/L | | 0.997 (0.992, 1.002) | 0.248 | 1.001 (1.000, 1.002) | 0.110 |
| Platelet, 10^9^/L | | 1.001 (1.000, 1.003) | 0.149 | 1.000 (0.994, 1.007) | 0.989 |
| Total protein, g/L | | 0.992 (0.978, 1.007) | 0.290 | 1.001 (0.999, 1.003) | 0.263 |
| ALT, U/L | | 1.005 (1.000, 1.010) | 0.051 | 1.002 (0.997, 1.007) | 0.433 |
| ALP, U/L | | 1.002 (1.001, 1.005) | 0.010 | 1.003 (1.000, 1.005) | 0.033 |
| γ-GGT, U/L | | 1.001 (1.000, 1.001) | 0.002 | 1.002 (1.001, 1.003) | 0.004 |

PSM, propensity score matching; LR, liver resection; PA, postoperative adjuvant; TACE, transcatheter arterial chemoembolization; LT, lenvatinib plus tislelizumab; HBV, hepatitis B virus; HCV, hepatitis C virus; AH, alcoholic hepatitis; AFP, alpha-fetoprotein; BCLC, Barcelona Clinic Liver Cancer; ES, Edmondson-Steiner; MVI, microvascular invasion; ALBI, albumin-bilirubin; ALT, aspartate aminotransferase; ALP, alkaline phosphatase; γ-GGT, gamma-glutamyl transferase.

Supplementary table 4.B Univariate Cox regression analysis of OS in the entire cohort and PSM cohort

| Characteristics | | the Entire cohort | | the PSM cohort | |
| --- | --- | --- | --- | --- | --- |
|  |  | HR (95% CI) | P | HR (95% CI) | P |
| Type of treatment | LR | Reference |  | Reference |  |
|  | PA-TACE | 0.225 (0.157, 0.323) | <0.001 | 0.172 (0.107, 0.275) | <0.001 |
|  | PA-LT | 0.270 (0.156, 0.467) | <0.001 | 0.305 (0.163, 0.569) | <0.001 |
| Age, yr (≤ 56 vs > 56) | | 1.078 (0.775, 1.498) | 0.656 | 0.990 (0.659, 1.487) | 0.962 |
| Gender (Male vs Female) | | 1.608 (0.890, 2.905) | 0.115 | 1.262 (0.582, 2.732) | 0.556 |
| Hepatitis | No hepatitis | Reference | - | Reference | - |
|  | HBV | 0.825 (0.495, 1.377) | 0.462 | 0.661 (0.267, 1.635) | 0.370 |
|  | HCV | 0.886 (0.404, 1.940) | 0.762 | 0.497 (0.143, 1.724) | 0.271 |
|  | AH | 0.732 (0.270, 1.985) | 0.540 | - | 0.965 |
| Liver Cirrhosis (Positive vs Negative) | | 1.000 (0.700, 1.429) | 0.998 | 0.905 (0.569, 1.440) | 0.674 |
| AFP, ng/ml (≥ 200 vs < 200) | | 1.665 (1.200, 2.310) | 0.002 | 1.526 (1.016, 2.292) | 0.042 |
| Tumor diameter, cm (≥ 5 vs < 5) | | 2.192 (1.567, 3.067) | <0.001 | 2.132 (1.414, 3.189) | <0.001 |
| Tumor number (Multiple vs Single) | | 1.428 (0.965, 2.115) | 0.075 | 0.987 (0.581, 1.676) | 0.961 |
| BCLC grade (B vs 0+A) | | 1.999 (1.257, 3.177) | 0.003 | 1.568 (0.813, 3.026) | 0.180 |
| ES (3/4 vs 1/2） | | 2.086 (1.486, 2.926) | <0.001 | 1.983 (1.295, 3.037) | 0.002 |
| MVI (Positive vs Negative) | | 2.328 (1.607, 3.372) | <0.001 | 2.622 (1.676, 4.100) | <0.001 |
| ALBI grade (2 vs 1) | | 1.162 (0.822, 1.643) | 0.392 | 1.265 (0.816, 1.961) | 0.293 |
| Child-pugh score (6 vs 5) | | 1.126 (0.709, 1.790) | 0.615 | 1.108 (0.591, 2.080) | 0.749 |
| Resection pattern (Nonanatomic vs Anatomic) | | 0.597 (0.397, 0.898) | 0.013 | 0.569 (0.341, 0.952) | 0.032 |
| Blood transfusion (Yes vs No) | | 1.081 (0.697, 1.677) | 0.729 | 1.175 (0.782, 1.765) | 0.438 |
| Blood loss, ml | | 1.000 (1.000, 1.000) | 0.329 | 1.000 (0.999, 1.001) | 0.788 |
| Operative time, min | | 1.001 (1.000, 1.003) | 0.061 | 0.689 (0.346, 1.373) | 0.289 |
| Hemoglobin, g/L | | 1.002 (0.994, 1.010) | 0.697 | 1.002 (1.000, 1.011) | 0.012 |
| Platelet, 10^9^/L | | 1.003 (1.000, 1.005) | 0.010 | 1.004 (0.994, 1.015) | 0.421 |
| Total protein, g/L | | 1.004 (0.980, 1.028) | 0.767 | 1.004 (1.000, 1.007) | 0.034 |
| ALT, U/L | | 1.003 (0.944, 1.011) | 0.547 | 1.005 (0.996, 1.014) | 0.297 |
| ALP, U/L | | 1.003 (1.001, 1.005) | 0.034 | 1.003 (1.000, 1.006) | 0.056 |
| γ-GGT, U/L | | 1.001 (1.000, 1.002) | 0.012 | 1.003 (1.001, 1.005) | 0.001 |

PSM, propensity score matching; LR, liver resection; PA, postoperative adjuvant; TACE, transcatheter arterial chemoembolization; LT, lenvatinib plus tislelizumab; HBV, hepatitis B virus; HCV, hepatitis C virus; AH, alcoholic hepatitis; AFP, alpha-fetoprotein; BCLC, Barcelona Clinic Liver Cancer; ES, Edmondson-Steiner; MVI, microvascular invasion; ALBI, albumin-bilirubin; ALT, aspartate aminotransferase; ALP, alkaline phosphatase; γ-GGT, gamma-glutamyl transferase.

Supplementary table 5 Summary of adverse events in PA-TACE and PA-LT groups

| Adverse events | the Entire cohort | | | | the PSM cohort | | | |
| --- | --- | --- | --- | --- | --- | --- | --- | --- |
|  | Grade 1–2, *n* (%) | | Grade 3, *n* (%) | | Grade 1–2, *n* (%) | | Grade 3, *n* (%) | |
|  | PA-TACE  (n=326) | PA-LT  (n=90) | PA-TACE  (n=326) | PA-LT  (n=90) | PA-TACE  (n=223) | PA-LT  (n=75) | PA-TACE  (n=223) | PA-LT  (n=75) |
| Pain | 132 (40.49) | 0 (0.00) | 0 (0.00) | 0 (0.00) | 91 (40.80) | 0 (0.00) | 0 (0.00) | 0 (0.00) |
| Elevated transaminases | 124 (38.04) | 20 (22.22) | 5 (1.53) | 0 (0.00) | 83 (37.22) | 17 (26.67) | 4 (1.79) | 0 (0.00) |
| Nausea/vomiting | 102 (31.29) | 0 (0.00) | 0 (0.00) | 0 (0.00) | 64 (29.15) | 0 (0.00) | 0 (0.00) | 0 (0.00) |
| Fever | 95 (29.19) | 4 (4.44) | 7 (2.15) | 0 (0.00) | 60 (26.91) | 3 (4.00) | 5 (2.24) | 0 (0.00) |
| Thrombocytopenia | 90 (27.61) | 16 (17.78) | 0 (0.00) | 0 (0.00) | 60 (26.91) | 13 (17.33) | 0 (0.00) | 0 (0.00) |
| Neutropenia | 88 (26.99) | 18 (20.00) | 5 (1.53) | 0 (0.00) | 60 (26.91) | 15 (20.00) | 4 (1.79) | 0 (0.00) |
| Elevated total bilirubin | 82 (25.15) | 12 (13.33) | 7 (2.15) | 0 (0.00) | 58 (26.01) | 10 (13.33) | 5 (2.24) | 0 (0.00) |
| Decreased albumin | 74 (22.70) | 8 (8.89) | 4 (1.23) | 0 (0.00) | 52 (23.32) | 6 (8.00) | 3 (1.35) | 0 (0.00) |
| Gastrointestinal hemorrhage | 45 (13.80) | 6 (6.67) | 7 (2.15) | 3 (3.33) | 32 (14.35) | 5 (6.67) | 5 (2.24) | 3 (4.00) |
| Diarrhea | 37 (11.35) | 9 (10.00) | 0 (0.00) | 0 (0.00) | 26 (11.66) | 7 (9.33) | 0 (0.00) | 0 (0.00) |
| Anemia | 34 (10.42) | 4 (4.44) | 0 (0.00) | 0 (0.00) | 24 (10.76) | 0 (0.00) | 0 (0.00) | 0 (0.00) |
| Fatigue | 22 (6.75) | 16 (17.78) | 0 (0.00) | 0 (0.00) | 14 (6.28) | 13 (17.33) | 0 (0.00) | 0 (0.00) |
| Hypertension | 13 (3.99) | 25 (27.78) | 0 (0.00) | 2 (2.22) | 8 (3.59) | 23 (30.67) | 0 (0.00) | 1 (1.33) |
| Proteinuria | 0 (0.00) | 5 (5.56) | 0 (0.00) | 0 (0.00) | 0 (0.00) | 3 (4.00) | 0 (0.00) | 0 (0.00) |
| Rash | 0 (0.00) | 4 (4.44) | 0 (0.00) | 0 (0.00) | 0 (0.00) | 3 (4.00) | 0 (0.00) | 0 (0.00) |
| Hand-foot skin reactions | 0 (0.00) | 9 (10.00) | 0 (0.00) | 2 (2.22) | 0 (0.00) | 8 (10.67) | 0 (0.00) | 2 (2.67) |
| Hypothyroidism | 0 (0.00) | 6 (6.67) | 0 (0.00) | 0 (0.00) | 0 (0.00) | 5 (6.67) | 0 (0.00) | 0 (0.00) |

PA, postoperative adjuvant; TACE, transcatheter arterial chemoembolization; LT, lenvatinib plus tislelizumab.

Note: The adverse events related with PA-TACE and PA-LT were graded according to Common Terminology Criteria Adverse Events (CTCAE) Version 5.0.

Supplementary Figure 1 Comparison of standardized mean differences before and after weighting


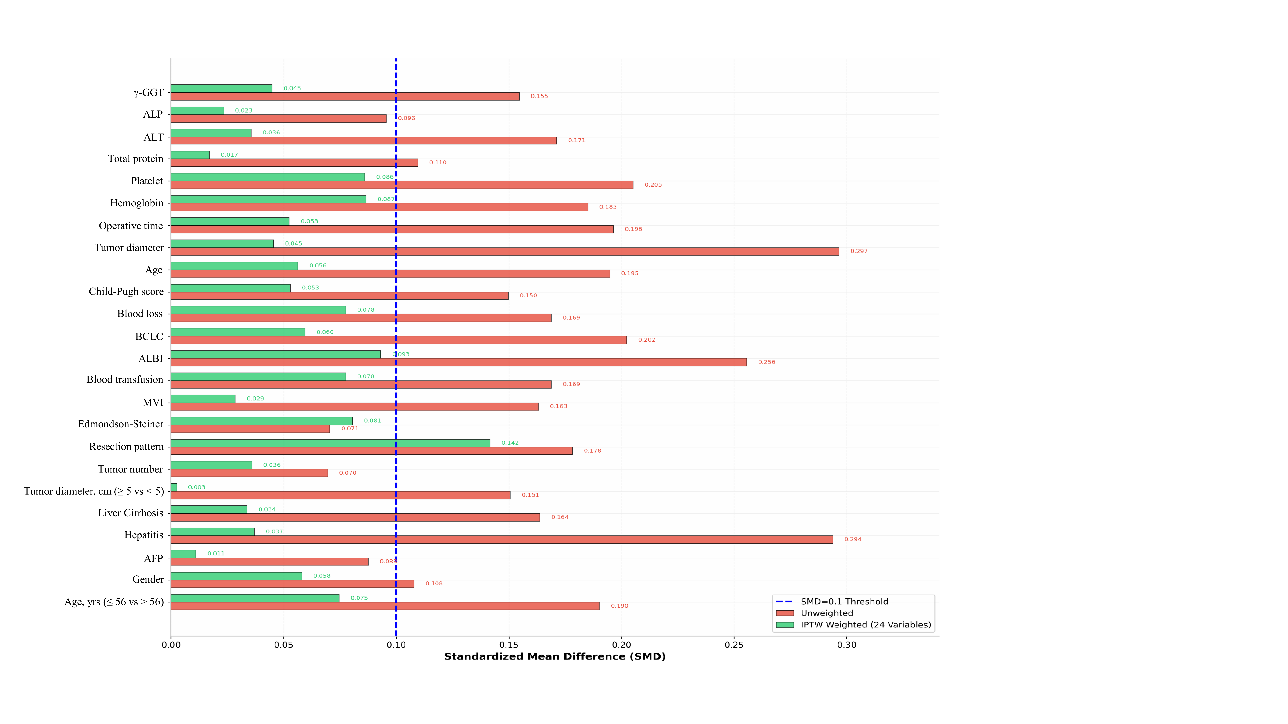


γ-GGT, gamma-glutamyl transferase; ALP, alkaline phosphatase; ALT, aspartate aminotransferase; BCLC, Barcelona Clinic Liver Cancer; ALBI, albumin-bilirubin; MVI, microvascular invasion; AFP, alpha-fetoprotein;
